# Outcomes of standardized drain-free abdominoplasty after massive weight loss: A retrospective analysis of 2533 post-bariatric patients

**DOI:** 10.1016/j.jpra.2026.04.005

**Published:** 2026-04-24

**Authors:** Ciro Borriello, Simona Barone, Gerardo Borriello, Maria Spagnuolo, Vincenzo Pilone, Antonio Vitiello

**Affiliations:** a “Clinica Cobellis”, Salerno, Italy; b Medicine and Surgery Department, Naples “Federico II” University, AOU “Federico II”, Naples, Italy; c Public Health Department, Naples “Federico II” University, AOU “Federico II”, Naples, Italy; d Advanced Biomedical Sciences Department, Naples “Federico II” University, AOU “Federico II”, Naples, Italy

**Keywords:** Post-bariatric abdominoplasty, Progressive tension sutures, Drain-free technique, Seroma prevention, Body contouring outcomes

## Abstract

This retrospective study evaluates the outcomes of 2533 standardized drain‑free abdominoplasties performed after massive weight loss, representing one of the largest series reported in post‑bariatric body‑contouring surgery. All procedures were carried out by the same surgical team across two high‑volume centers using a uniform protocol based on progressive tension sutures (PTS), meticulous flap handling, and structured postoperative management. The cohort reflected typical post‑bariatric characteristics, with a strong female predominance, mean BMI in the obese range, and a short average hospital stay. Most patients underwent a low transverse “smile” incision, while the fleur‑de‑lis approach was reserved for combined vertical and horizontal laxity. Diabetes was present in 5.4% of patients and evenly distributed between BMI groups. Overall complication rates were acceptable, supporting the safety and reproducibility of a drain‑free technique in this challenging population. However, BMI emerged as a major determinant of postoperative morbidity. Patients with BMI ≥30 kg/m² experienced significantly higher rates of seroma, umbilical necrosis, wound dehiscence, hematoma requiring re‑intervention, and surgical site infection. These findings highlight the detrimental effects of excess adiposity on perfusion, lymphatic drainage, and wound stability. The similar prevalence of diabetes across groups suggests that BMI itself, rather than unequal metabolic burden, primarily drives the observed differences, although diabetes remains an additional contributor to impaired healing.

## Introduction

Global bariatric and metabolic surgery continues to grow significantly, with the latest IFSO registry reporting over 502,000 procedures across 24 countries and 2 regional registries. Sleeve gastrectomy remains the most common primary procedure worldwide, while Roux-en-Y gastric bypass dominates revisional cases.^1^ Massive weight loss after bariatric surgery is a well‑recognized risk factor for postoperative fluid collections following body‑contouring procedures.^2^ The profound reduction in subcutaneous fat often leaves patients with extensive dead space and compromised lymphatic drainage. These anatomical changes predispose the abdominal flap to seroma formation, even when meticulous surgical technique is applied. In addition, the quality of the residual tissues is frequently poor, with reduced elasticity and altered vascularity. Such factors further impair fluid resorption and wound healing. Bariatric patients may also present with nutritional deficiencies^3^ that negatively affect collagen synthesis and inflammatory responses. These systemic vulnerabilities contribute to higher rates of seroma, hematoma, and wound dehiscence.^4^^,^^5^

Abdominoplasty evolved from early dermolipectomies with transverse, vertical, or combined excisions,^6^, ^7^, ^8^ progressing to more refined contouring approaches such as Vernon’s low transverse technique with umbilical transposition,^9^ Callia’s low inguinal incision,^10^ and Pitanguy’s widely adopted low transverse abdominoplasty with extensive undermining and muscle tightening.^11^ Subsequent innovations included Regnault’s “W” incision,^12^ Planas’ “vest‑over‑pants” technique,^13^ and circumferential belt lipectomy for massive pannus.^14^ The introduction of suction‑assisted lipectomy revolutionized abdominal contouring, enabling combined lipo‑abdominoplasty^15^ and later limited‑dissection techniques by Saldanha^16^ and Avelar.^17^

Progressive tension sutures (PTS) have been proposed as an effective strategy to reduce seroma formation and eliminate the need for drains.^18^

The aim of this study was to retrospectively evaluate the clinical outcomes, complication rates, and safety of standardized, drain-free post-bariatric abdominoplasty performed after massive weight loss, with particular attention to the influence of patient characteristics, incision patterns, and enhanced recovery protocols.

## Materials and methods

A retrospective cohort study was conducted to evaluate the outcomes of post‑bariatric abdominoplasty procedures performed between February 2017 and January 2025 at two accredited surgical centres. All operations were performed by the same experienced surgical team, following a standardized operative technique and postoperative management protocol.

This retrospective study was designed, conducted, and reported in accordance with the STROBE (Strengthening the Reporting of Observational Studies in Epidemiology) guidelines for observational cohort studies, ensuring methodological rigor and transparent reporting of all study phases.^19^

The study population included patients who had previously undergone bariatric surgery—either laparoscopic sleeve gastrectomy or Roux‑en‑Y gastric bypass—and subsequently presented with redundant abdominal tissue requiring contouring surgery. Only patients who met strict inclusion criteria were selected for analysis. These criteria included:

• A documented history of bariatric surgery with stable weight maintained for at least 12 consecutive months prior to abdominoplasty.
• Absence of ongoing anticoagulant therapy and no history of coagulation disorders.
• Availability of complete preoperative, intraoperative, and postoperative data, including a minimum follow‑up of 6 months.

Patients were excluded if they underwent revisional abdominoplasty, required concomitant major surgical procedures (e.g., hernia repair, flank or thigh lift), experienced intraoperative complications necessitating drain placement, or had insufficient documentation for longitudinal follow‑up. Cases with early loss to follow‑up or incomplete clinical records were also excluded to ensure consistency. Patients at our practice are routinely encouraged to stop smoking or to drastically reduce the consumption of cigarettes.

Collected variables included demographic data (age, sex), type of bariatric procedure, abdominoplasty technique (conventional vs. fleur-de-lis), length of hospital stay, and postoperative complications. Postoperative complications of interest included seroma, umbilical necrosis, wound dehiscence, postoperative bleeding or hematoma requiring surgical re-intervention, venous thromboembolism, pulmonary embolism, and mortality. Seroma was diagnosed clinically and confirmed by ultrasound when necessary. Umbilical necrosis and wound dehiscence were identified during routine wound assessments. Postoperative bleeding or hematoma was defined by clinical signs or a significant hemoglobin drop, with operative evacuation classified as a major event. Venous thromboembolism and pulmonary embolism were investigated only when clinically suspected, following standard diagnostic pathways.

For analytical purposes, the cohort was stratified into two groups based on preoperative BMI: patients with BMI <30 kg/m² and those with BMI ≥30 kg/m². Outcomes, complication rates, and perioperative characteristics were compared between the two groups to evaluate the impact of BMI on postoperative results.

### Statistical analysis

Continuous variables were expressed as mean ± standard deviation and compared between groups using the student’s t-test after assessment of normality. Categorical variables, including the distribution of postoperative complications and the prevalence of diabetes, were analyzed using the chi‑square test or Fisher’s exact test when appropriate.

### Surgical technique

Incision choice correlated with BMI and skin laxity distribution: patients with BMI <30 kg/m² and predominantly horizontal laxity were more frequently candidates for the smile incision, whereas those with BMI >30–35 kg/m² or combined vertical and horizontal excess benefited from the fleur‑de‑lis approach. Preoperative markings included a low transverse incision within the suprapubic region and lateral extension toward the anterior iliac spines, with the upper excision limit determined by manual traction considering redundancy and previous scars. On the operating table, dissection proceeded above Scarpa’s fascia to 3–4 cm from the xiphoid, preserving the umbilical stalk. Rectus plication was performed when indicated. Progressive tension sutures were systematically placed between the flap and underlying fascia to eliminate dead space and reduce seroma risk, followed by layered closure and umbilical transposition. Postoperatively, calibrated compression garments, polyurethane foam, and elastic adhesive bandaging (Tensoplast®) were applied. Patients underwent blood tests the night of surgery and the following day, resumed oral nutrition as soon as tolerated, and were mobilized the morning after dressing change and application of a compression binder.

## Results

**Figure 1:**
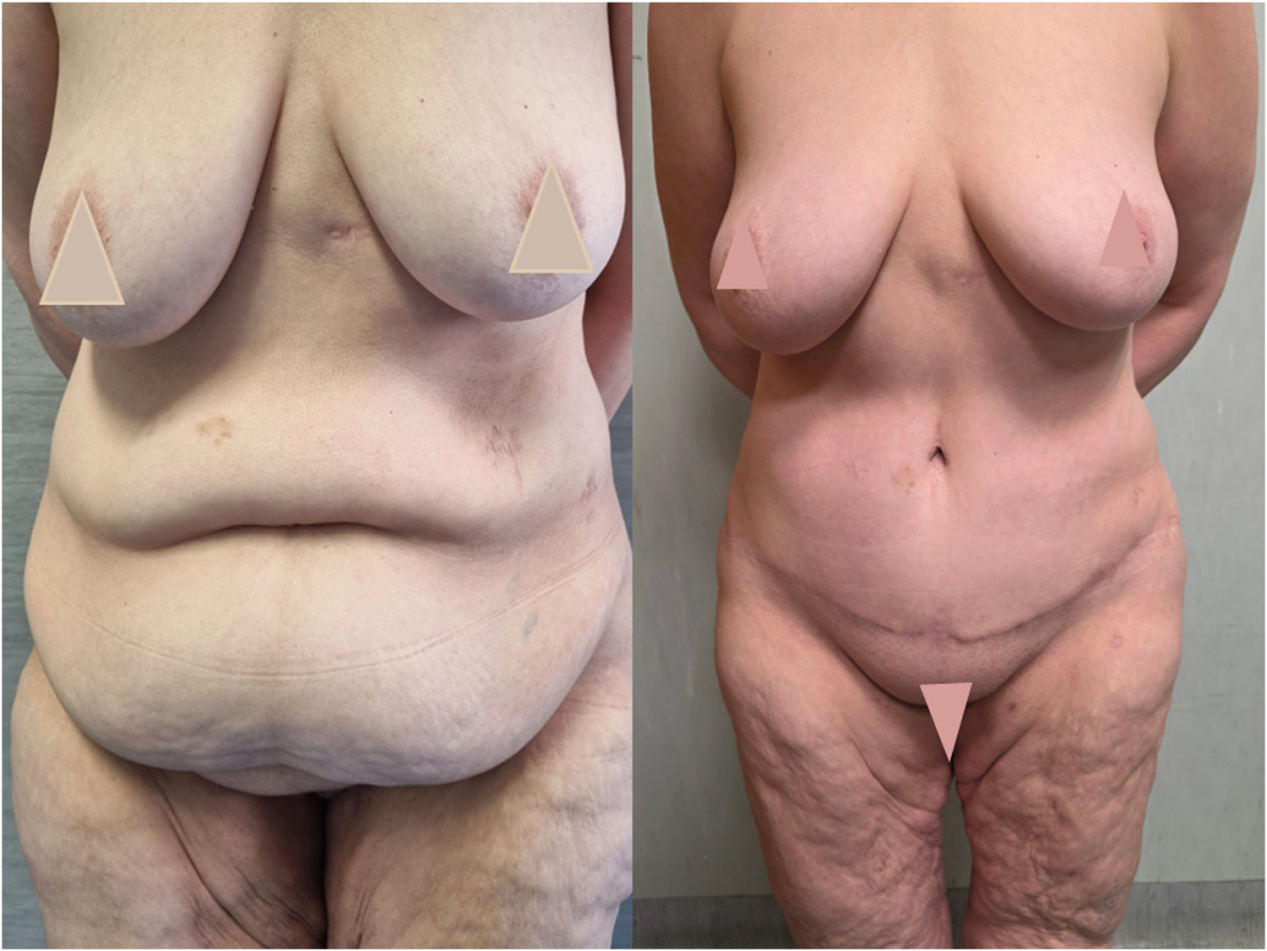
Marked improvement in abdominal contour following low‑transverse abdominoplasty performed with progressive tension sutures.

**Figure 2:**
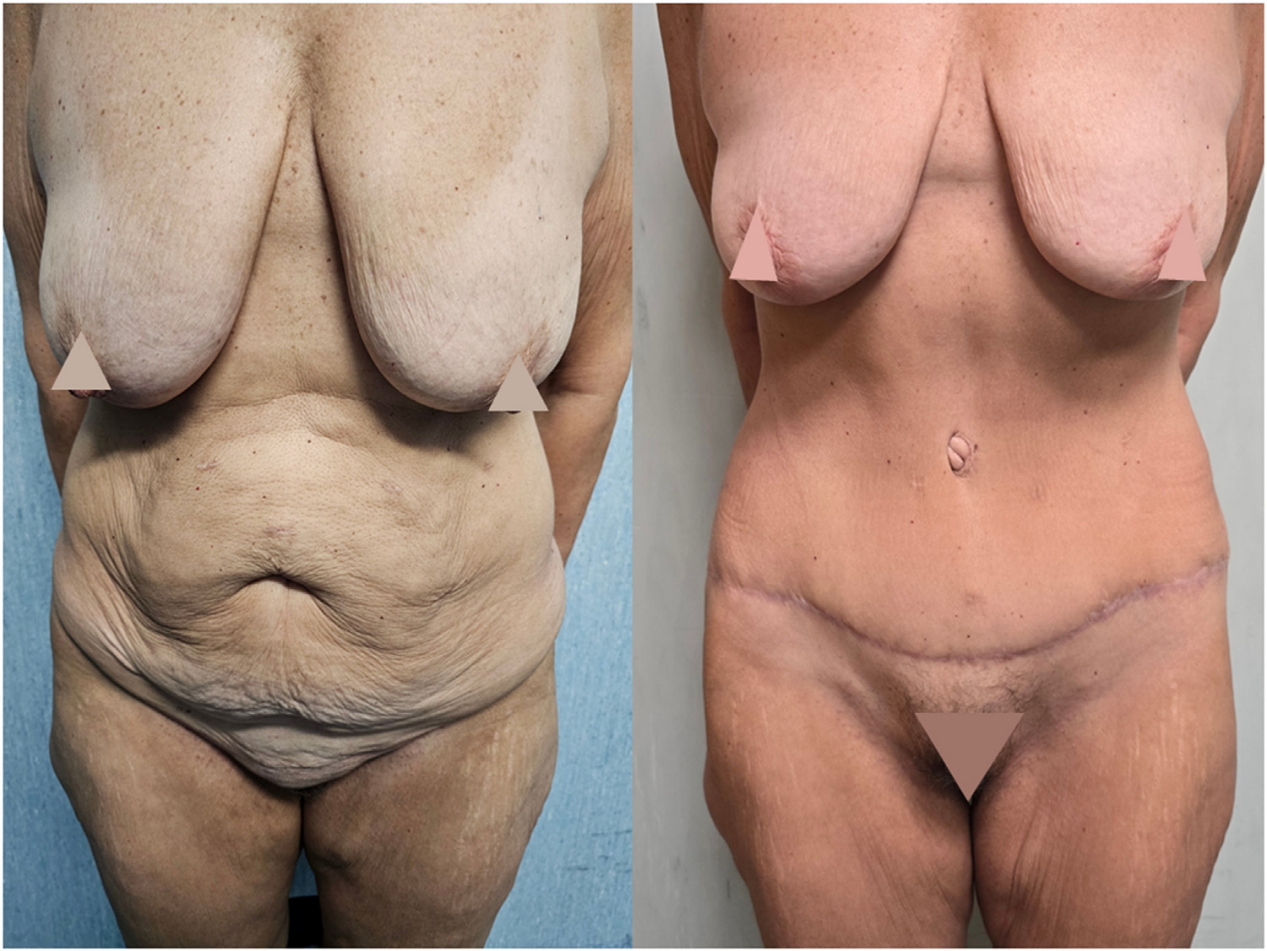
Before‑and‑after comparison in a female patient following low‑transverse incision.

**Figure 3:**
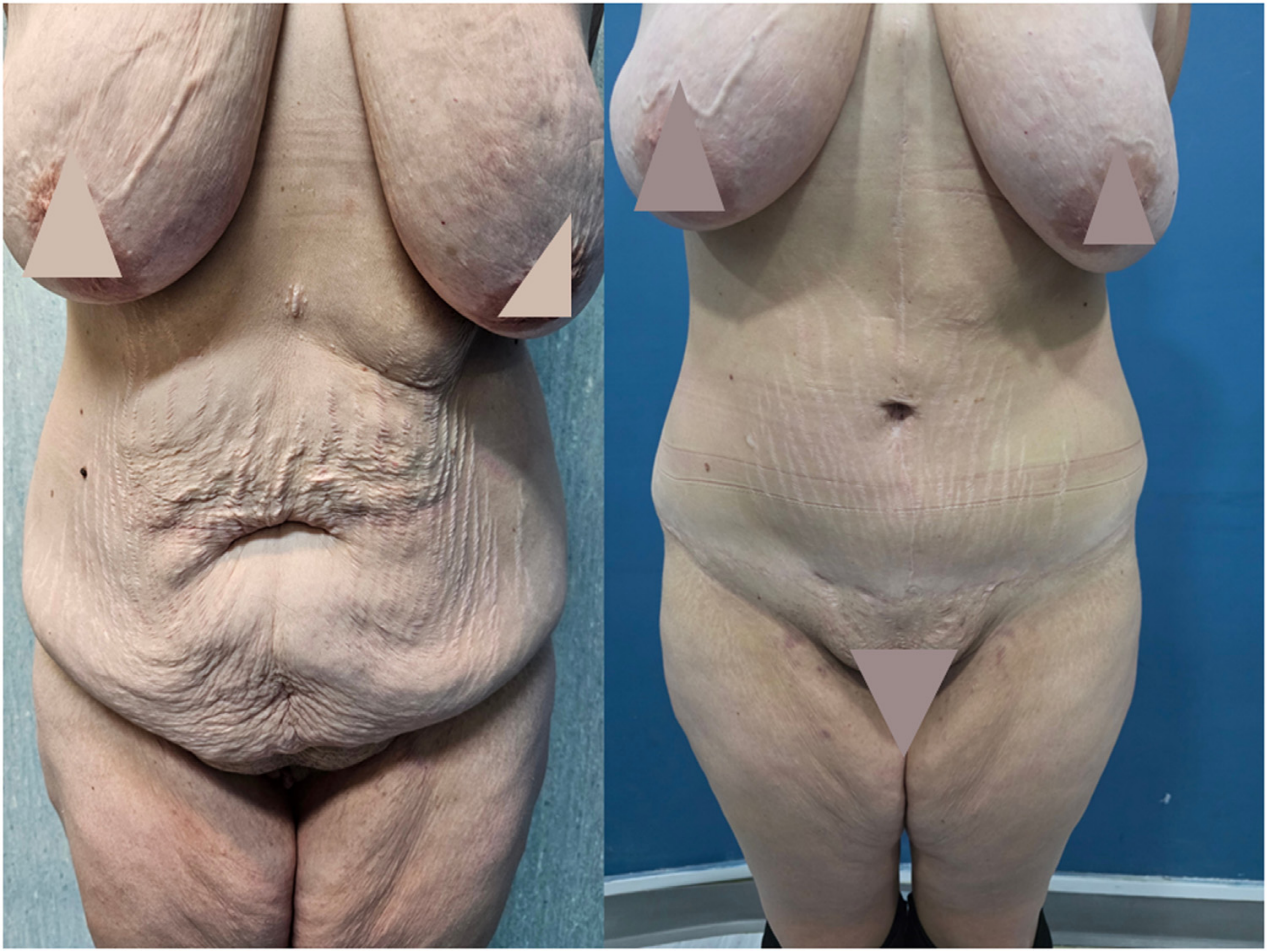
Before‑and‑after comparison in a female patient following fleur-de-lis abdominoplasty.

**Figure 4:**
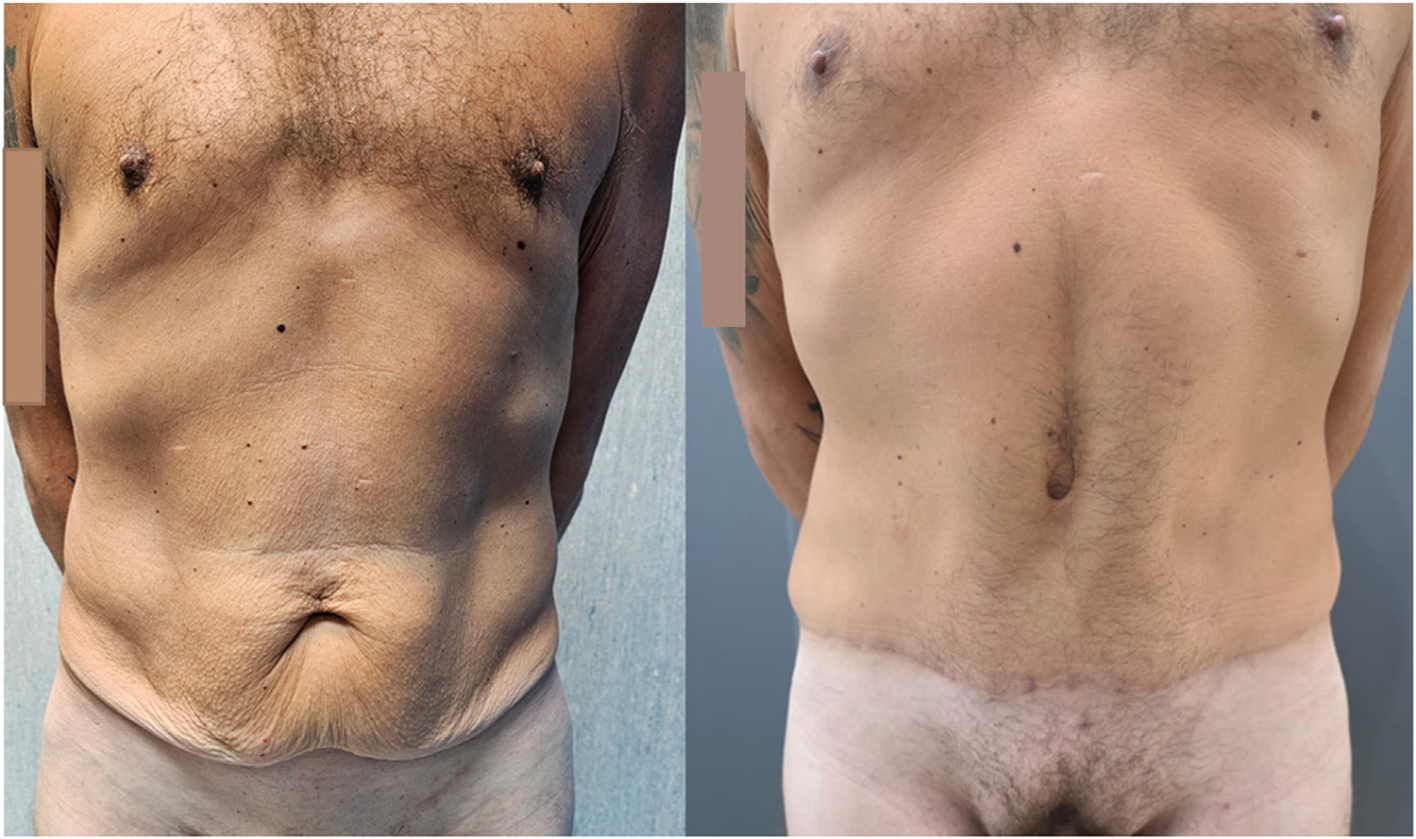
Before‑and‑after comparison in a male patient.

A total of 2533 abdominoplasties were performed during the study period, comprising 2099 females (82.9%) and 434 males (17.1%). The mean age was 42 ± 2.65 years, and the mean BMI was 34.8 ± 4.2 kg/m². The average hospital stay was 2.65 ± 0.88 days. Diabetes was present in 137 patients (5.4%), of whom 65 (5.3%) belonged to the BMI <30 kg/m² cohort and 72 (5.5%) to the BMI ≥30 kg/m² cohort; this difference was not statistically significant (*p* = 0.84). Regarding surgical technique, 1596 procedures (63%) were performed using a low transverse “smile” incision, while 937 procedures (37%) required a fleur‑de‑lis pattern to address circumferential skin redundancy (Figure 1, Figure 2, Figure 3, Figure 4, Table 1).

**Table 1:** Baseline characteristics of the study population.

*Variable*	*Value*
*Total procedures*	*2533*
*Female sex*	*2099 (82.9%)*
*Mean age (years)*	*42 ± 2.65*
*Mean BMI (kg/m²)*	*34.8 ± 4.2*
*Hospital stay (days)*	*2.65 ± 0.88*
*Diabetes mellitus*	*137 (5.4%)*
• *BMI <30 kg/m²*	*65 (5.3%)*
• *BMI ≥30 kg/m²*	*72 (5.5%)*
*Surgical technique*	
• *Low transverse “smile” incision*	*1596 (63%)*
• *Fleur-de-lis incision*	*937 (37%)*

Regarding cohorts (Table 2), 1216 patients had a BMI <30 kg/m² (48.0%) and 1317 patients had a BMI ≥30 kg/m² (52.0%). Complications were significantly more frequent in the higher‑BMI cohort. Seroma occurred in 18 patients (1.5%) in the BMI <30 group versus 58 patients (4.4%) in the BMI ≥30 group (*p* = 0.0000017). Umbilical necrosis was observed in 21 patients (1.7%) with BMI <30 and in 55 patients (4.2%) with BMI ≥30 (*p* = 0.00011). Wound dehiscence occurred in 29 patients (2.4%) in the BMI <30 cohort compared with 72 patients (5.5%) in the BMI ≥30 cohort (*p* = 0.000014). Postoperative bleeding or hematoma requiring surgical re‑intervention was recorded in 27 patients (2.2%) with BMI <30 and in 67 patients (5.1%) with BMI ≥30 (*p* = 0.000036). Surgical site infections (SSI) occurred in 65 patients overall, including 24 patients (2.0%) in the BMI <30 group and 41 patients (3.1%) in the BMI ≥30 group (*p* = 0.047).

**Table 2:** Distribution of postoperative complications stratified by BMI category.

Complication	BMI <30 (*n* = 1216)	BMI ≥30 (*n* = 1317)	*p*-value (χ²)
Seroma	18 (1.5%)	58 (4.4%)	*p* = 0.0000017
Umbilical necrosis	21 (1.7%)	55 (4.2%)	*p* = 0.00011
Wound dehiscence	29 (2.4%)	72 (5.5%)	*p* = 0.000014
Bleeding/hematoma	27 (2.2%)	67 (5.1%)	*p* = 0.000036
SSI	24 (2.0%)	41 (3.1%)	*p* = 0.047
Diabetes prevalence	65 (5.3%)	72 (5.5%)	*p* = 0.84

## Discussion

The surgical approach to abdominoplasty, particularly in post-bariatric patients, has evolved substantially in recent years. Increasing evidence supports the safety and efficacy of drain-free techniques, largely due to the introduction of progressive tension sutures (PTS).^6^ By anchoring the abdominal flap to the underlying fascia, PTS eliminate dead space, reduce shear forces, and distribute tension key factors in minimizing seroma formation.

Pollock et al.^20^ in a retrospective review of 597 consecutive PTS abdominoplasties over 12 years reported an overall local complication rate of 4.2%, with seroma occurring in only 0.1% of cases (one patient).

A meta-analysis on PTS^21^ included 1255 patients undergoing abdominoplasty, predominantly women with a mean age ranging from 37 to 45 years and demonstrated that progressive tension sutures significantly reduced seroma rates compared with drains alone (OR 0.36; *p* = 0.002). Similarly, in a prospective, randomized, double-blind clinical trial, Andrades et al.^22^ demonstrated that progressive tension sutures significantly reduced postoperative drain output and shortened the duration of drain use compared with standard closure.

On the contrary, a recent systematic review by Arkoubi et al. evaluated five prospective trials involving 130 abdominoplasty patients and found no significant evidence that postoperative compression garments reduce seroma formation or improve ventilatory function, subcutaneous edema, or overall recovery.^23^

Consistently with these results, another review^24^ analyzing five studies encompassing 130 abdominoplasty patients found no strong evidence that postoperative compression garments reduce seroma, edema, or other complications.

Surprisingly, a randomized clinical trial^25^ of 32 women undergoing abdominoplasty found that patients who did not wear a postoperative compression garment developed less subcutaneous edema after 24–35 days compared with those who wore one. Seroma rates were similar between groups, indicating that compression garments did not provide a protective effect and may even delay edema resolution.

However, massive weight loss following bariatric surgery is a well-recognized risk factor for postoperative fluid collections after abdominoplasty,^26^ and this risk appears to be even greater compared to weight loss achieved through nonsurgical methods.^27^

For these reasons, several strategies—such as the use of coagulation devices,^28^ fibrin sealants,^29^ and BMI-based eligibility thresholds^30^—have been proposed to reduce dead space and fluid-related complications in this challenging patient population. Nevertheless, we elected to rely on our standardized drain-free technique, which has consistently demonstrated safety and reproducibility in our hands.

Our cohort reflected the typical abdominoplasty population, with a strong female predominance and BMI values in the obese range. Despite elevated BMI and a 9.6% prevalence of diabetes, patients generally tolerated surgery well, as indicated by the short mean hospital stay. The distribution of techniques—favoring the low transverse incision and reserving the fleur‑de‑lis approach for more complex circumferential laxity—illustrated the heterogeneity of abdominal deformities encountered.

Our results indicate that abdominoplasty with progressive tension sutures maintains overall acceptable complication rates, but they also show a clear and consistent vulnerability in patients with elevated BMI. Every major wound-related event—seroma, necrosis, dehiscence, hematoma, and SSI—occurs significantly more often in the BMI ≥ 30 kg/m² cohort, highlighting how excess adipose tissue compromises perfusion, lymphatic drainage, and mechanical stability of the wound. The similar prevalence of diabetes across groups confirms that BMI itself, rather than unequal metabolic burden, is driving most of the observed differences, although diabetes remains a recognized co-factor for impaired healing. Taken together, these findings clarify that abdominoplasty with PTS is safe across a broad population, but outcomes are strongly influenced by patient-related risk factors, particularly high BMI and metabolic comorbidities, which should guide preoperative counseling and perioperative optimization.

### Strengths and limitations

This study benefits from a large sample size, a uniform surgical technique, and consistent postoperative management across two high-volume centers. Its retrospective design and reliance on available documentation, however, may limit the precision of complication reporting. A potential selection bias arises from the exclusion of patients who required intraoperative drain placement. Because drains are typically used in cases with greater tissue dissection, higher intraoperative bleeding, or increased perceived risk of fluid accumulation, omitting these patients may have led to an underestimation of the true complication rate. Individuals requiring drains could reasonably be expected to have a higher likelihood of postoperative issues such as seroma, hematoma, or wound complications.

## Conclusion

This large retrospective series shows that standardized drain-free abdominoplasty after massive weight loss yields an acceptable rate of postoperative complications, even in patients with elevated BMI or diabetes. The consistent use of progressive tension sutures, meticulous flap handling, and a structured postoperative pathway appears to limit the incidence of seroma, hematoma, and wound-healing issues. At the same time, the marked increase in complications among patients with BMI ≥30 kg/m² confirms that excess adiposity remains a major risk factor for surgical morbidity. Overall, these findings support the safety and reproducibility of a no-drain protocol, while underscoring the importance of careful risk stratification in patients with higher BMI or metabolic comorbidities.

## Funding

None.

## Declaration of Competing Interest

None declared.
